# A novel imaging scoring method for identifying facial canal dehiscence: an ultra-high-resolution CT study

**DOI:** 10.1007/s00330-022-09231-2

**Published:** 2022-11-15

**Authors:** Ruowei Tang, Zhengyu Zhang, Pengfei Zhao, Lei Zhao, Ning Xu, Hongxia Yin, Zhenghan Yang, Zhenchang Wang

**Affiliations:** Department of Radiology, Beijing Friendship Hospital, Capital Medical University, 95 Yong’an Road, Xicheng District, Beijing, 100050 People’s Republic of China

**Keywords:** Facial nerve, Tomography, X-ray computed, Otologic surgical procedures, Iatrogenic disease

## Abstract

**Objectives:**

Facial canal dehiscence (FCD), typically found in the tympanic segment, is a risk factor for facial nerve injury. An imaging scoring method was proposed to identify FCD based on ultra-high-resolution CT.

**Methods:**

Forty patients (21 females and 19 males, mean age 44.3 ± 17.4 years), whose tympanic facial canal (FC) was examined during otological surgery, were divided into the FCD group (*n* = 29) and the control group (*n* = 11) based on surgical findings. Imaging appearance of tympanic FC was scored 0–3: 0 = no evident bony covering, 1 = discontinuous bony covering with linear deficiency, 2 = discontinuous bony covering with dotted deficiency, and 3 = continuous bony covering. Both lateral and inferior walls were assigned a score as L_FCD_ and I_FCD_, respectively. An FCD score was calculated as L_FCD_ + I_FCD._ The diagnostic value of the FCD score was tested using the ROC curve.

**Results:**

The inter-observer agreement was moderate for the lateral wall (Cohen’s κ coefficient 0.416, 95% CI 0.193–0.639), and good for the inferior wall (Cohen’s κ coefficient 0.702, 95% CI 0.516–0.888). In the FCD group, the most common appearance for both walls was discontinuous bony covering with linear deficiency (L_FCD_ = 1, 22/29, 75.9%; I_FCD_ = 1, 15/29, 51.7%). An FCD score of less than 4 was associated with high sensitivity (0.82) and specificity (0.93) for identifying FCD, with an AUC of 0.928.

**Conclusions:**

Using the proposed scoring method, FCD score < 4 could identify FCD of the tympanic segment with high concordance with surgical findings.

**Key Points:**

*• Imaging appearance of the tympanic facial canal (FC) is divided into four types based on ultra-high-resolution CT images.*

*• The most common appearance of FC with facial canal dehiscence (FCD) is discontinuous bony covering with linear deficiency.*

*• An FCD score, consisting of scores of the lateral and inferior walls, less than 4 is highly indicative of FCD.*

**Supplementary Information:**

The online version contains supplementary material available at 10.1007/s00330-022-09231-2.

## Introduction

Facial nerve (FN) is highly vulnerable to iatrogenic injury during otologic surgery [1]. Facial canal dehiscence (FCD) is one of the risk factors for FN injury. In FCD, instead of the bony coverage, the nerve is covered by a delicate fibrous membrane [2] and is exposed to the surgery field, sometimes even prolapsing into the tympanic cavity. In the healthy population, the incidence of FCD in the tympanic segment can be as high as 51.2% [3]. In clinical studies, FCD was identified in 6–33.3% of patients with cholesteatoma or otitis media [4–7], while the detection rate was much higher in anatomical studies (25–57%) [8, 9]. During surgical maneuvers, such as drilling to remove granulation tissue, FCD increases the risk of FN paralysis and deteriorates pathological conditions such as chronic otitis media (COM) [10, 11]. Of all the intratemporal segments, the tympanic segment of the facial canal (FC) is most likely to be dehiscent [3].

Since FN injury can lead to severe complications, close monitoring is suggested for preoperative, intraoperative, and postoperative management [11]. Preoperative CT evaluation of FCD plays a key role in preventing iatrogenic injury. However, the bony coating of the tympanic FC is thin, surpassing the spatial capability of routinely used multislice computed tomography (MSCT). The reported sensitivity and specificity of MSCT in identifying FCD are 64.7% and 78.4%, respectively [4]. Therefore, the currently used MSCT devices in clinical practice may not reliably detect FCD [3, 12, 13]. Previous studies on FCD have described it as the absence or discontinuity of bony covering [14]; however, there is a paucity of studies describing the detailed imaging appearance of FCD in contemporary literature. Therefore, the use of a CT device with higher spatial resolution may provide a more in-depth characterization of the radiological appearance of FCD.

A recently developed ultra-high-resolution computed tomography (U-HRCT), with a spatial resolution of 0.1 mm, may be helpful in detecting the presence of FCD. Studies have demonstrated the capability of U-HRCT in delineating fine structures of the temporal bone, both in cadavers and in patients with otologic diseases [15–17]. Therefore, the aims of this study were (1) to describe the imaging appearance of the tympanic FC based on U-HRCT images, and (2) to propose a novel imaging scoring method to identify FCD, using surgical finding as the gold standard.

## Materials and methods

### Eligible participants

This retrospective study was performed at our tertiary center with approval from the local ethical committee (IRB: 2020-P2-061-02). Written informed consent was waived by the institutional review board. The inclusion criterion was patients from the Otolaryngology Department who underwent U-HRCT between October 2020 and January 2022 (*n* = 829). The exclusion criteria were: (1) patients who did not undergo otological surgery (*n* = 68), (2) patients whose tympanic FC was not probed during otological surgery (*n* = 715), and (3) severe motion artifacts on U-HRCT images (*n* = 6). Finally, patients whose tympanic FC was probed during otological surgery were included (*n* = 40), and clinical and imaging data of the included patients were reviewed. Based on surgical findings, patients with dehiscence in the tympanic FC were categorized as the FCD group, and those with intact tympanic FC were categorized as the control group.

### Imaging protocols

All patients underwent imaging examination of bilateral temporal bones using a U-HRCT scanner (Ultra3D, LargeV) at 100 kVp and 9.0 mA, with a field of view of 65 mm. The slice thickness and interval were both set at 0.1 mm. The exposure time was 20 s, and isotropic axial images that could be reformatted from any desired direction were acquired.

### Image analysis

To standardize the observation planes, the coronal position lines on the axial and sagittal sections were first adjusted perpendicular to the long axis of the tympanic segment of the FN. Then, on the acquired coronal images, the axial and sagittal position lines were rotated to bring them parallel to the lateral and superior semicircular canals, respectively. After the above adjustments, the standard observation planes in the axial, sagittal, and coronal planes were defined (Supplementary Figure 1).

**Fig. 1:**
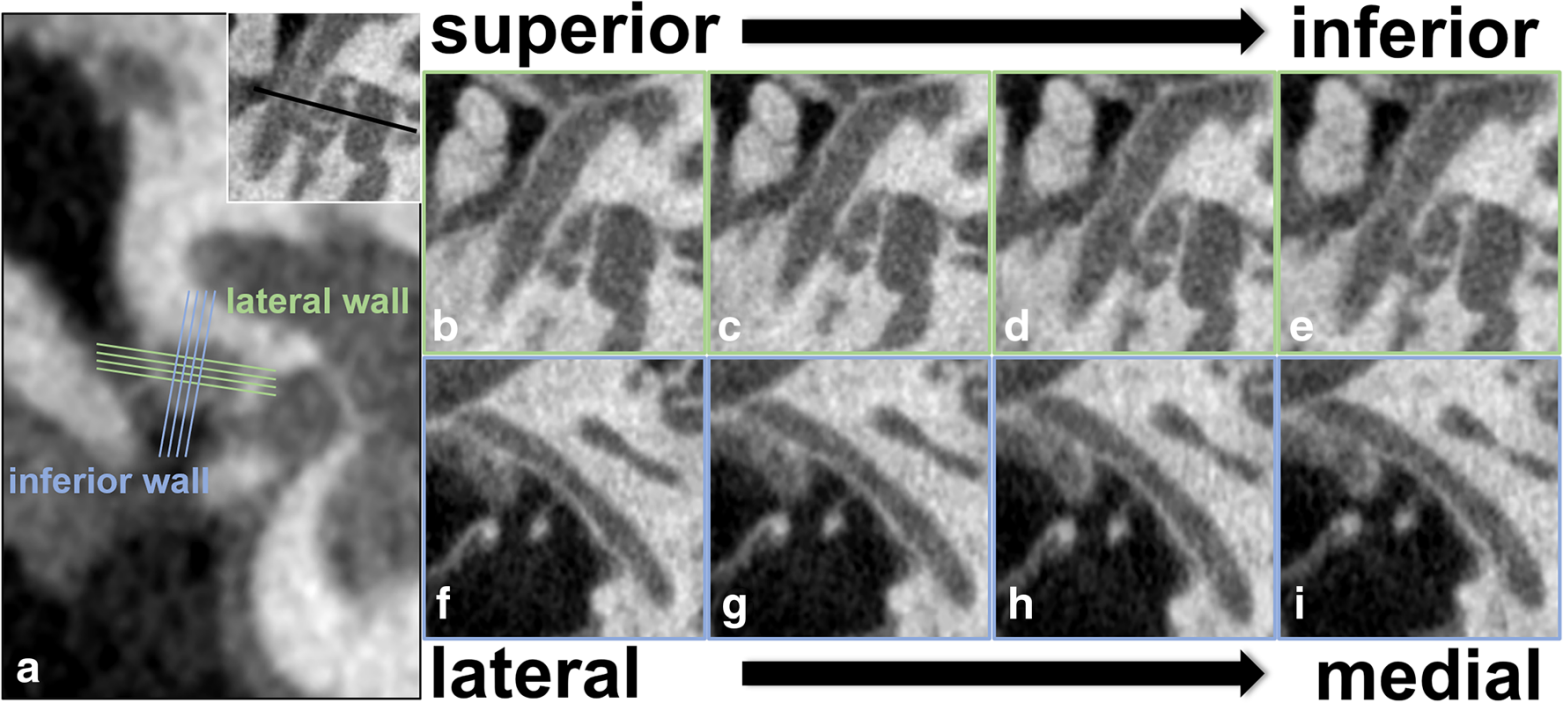
Standard observation slices for evaluation of imaging appearance of the tympanic facial canal. The lateral and inferior walls are evaluated on 4 consecutive slices on the axial and sagittal planes, respectively. The center of the nerve is found on the coronal image passing the midpoint of the stapes footplate (**a**). Imaging appearance of the lateral wall is evaluated on 2 slices superior and inferior to the nerve center (**b**–**e**), while the inferior wall is assessed on 2 slices lateral and medial to the nerve center (**f**–**i**)

Subsequently, standard observation slices were obtained. The coronal image passing the midpoint of the stapes footplate was chosen, on which the center of the tympanic FN was found. Two slices superior and 2 slices inferior to the FN center were used to evaluate the appearance of the lateral wall. Likewise, the integrity of the inferior wall was assessed on 2 slices lateral and 2 slices medial to the FN center. In other words, radiological evaluation of the lateral and inferior walls was performed on 4 consecutive slices on axial and sagittal planes, respectively (Fig. 1). In the anteroposterior direction, the portion between the semicanal of the tensor tympani and the pyramidal eminence was included for evaluation, since this was the portion hanging above the stapes, thus was of special importance during surgery.

**Fig. 2:**
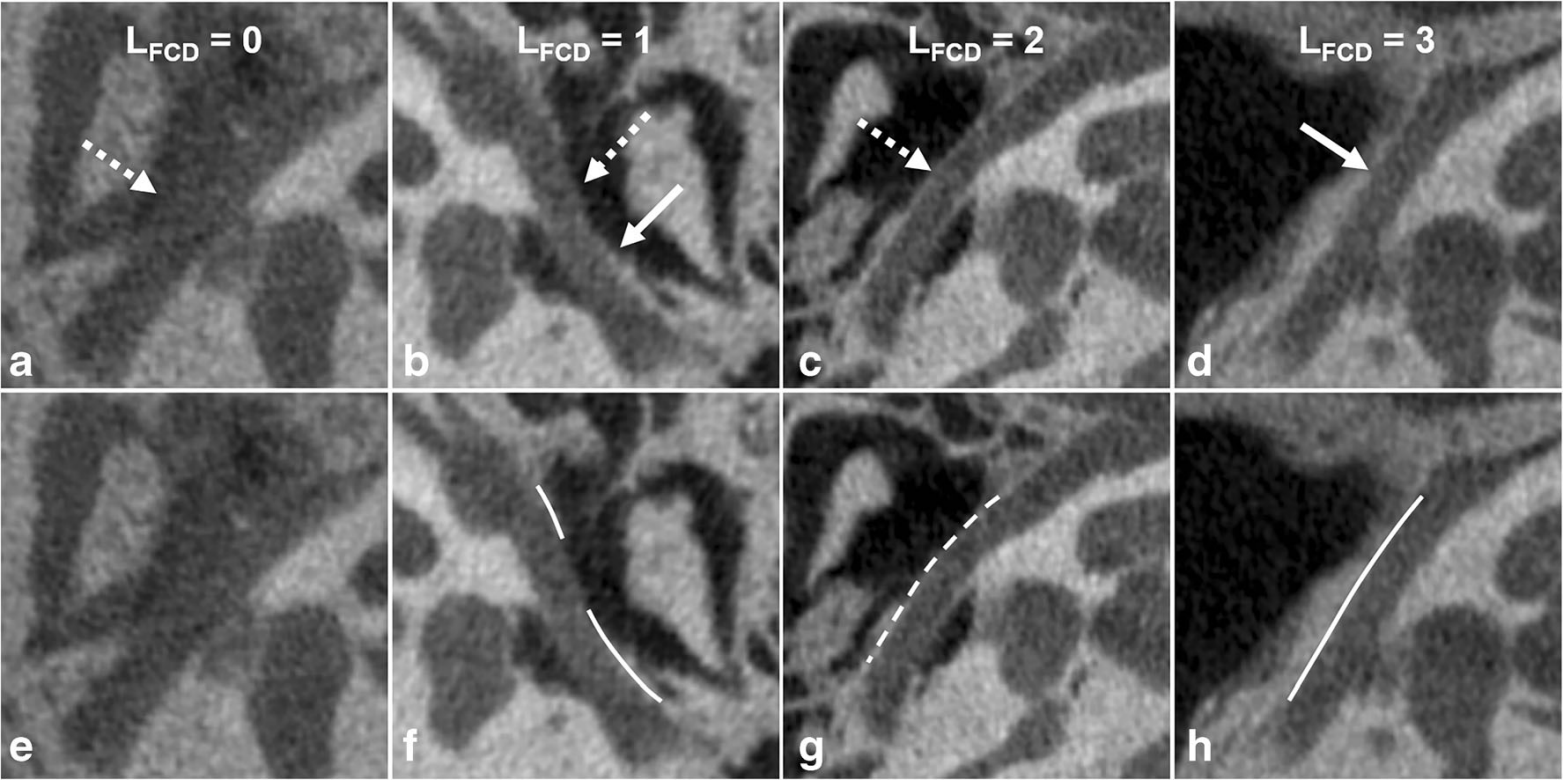
Score of the lateral wall (L_FCD_). L_FCD_ 0 = no evident bony covering (**a**, **e**); L_FCD_ 1 = discontinuous bony covering, linear deficiency (**b**, **f**); L_FCD_ 2 = discontinuous bony covering, dotted deficiency (**c**, **g**); and L_FCD_ 3 = continuous bony covering (**d**, **h**). Bony covering is indicated by solid arrows and lines, while bony deficiency is indicated by dashed ones

**Fig. 3:**
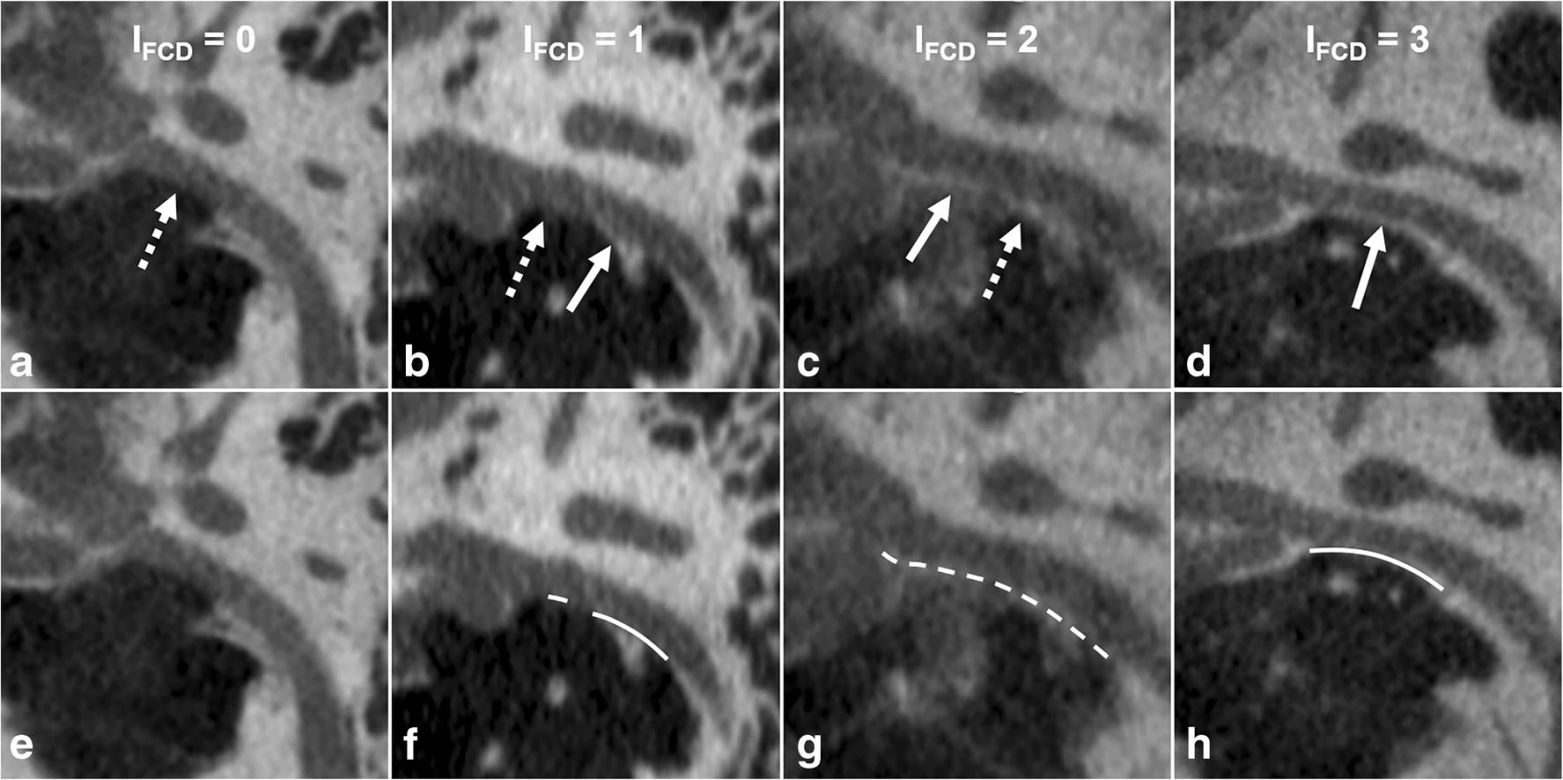
Score of the inferior wall (I_FCD_). I_FCD_ 0 = no evident bony covering (**a**, **e**); I_FCD_ 1 = discontinuous bony covering, linear deficiency (**b**, **f**); I_FCD_ 2 = discontinuous bony covering, dotted deficiency (**c**, **g**); and I_FCD_ 3 = continuous bony covering (**d**, **h**). Bony covering is indicated by solid arrows and lines, while bony deficiency is indicated by dashed ones

Imaging appearance of the lateral and inferior walls of the tympanic FC was first analyzed independently by 2 neuroradiologists (with 6- and 16-year experience in reviewing head and neck images, respectively), both of whom were blinded to the surgical findings. The lateral wall was evaluated on both the axial and coronal planes, whereas the inferior wall was analyzed on the sagittal and coronal sections. Imaging appearance of the tympanic FC was scored and categorized into the following 4 types: score 0 = no evident bony covering, score 1 = discontinuous bony covering with linear deficiency, score 2 = discontinuous bony covering with dotted deficiency, and score 3 = continuous bony covering. The scores assigned for lateral and inferior walls were referred to as L_FCD_ and I_FCD_, respectively (Figs. 2 and 3).

Then, after their independent analyses, the observers, still blinded to the surgical findings, re-evaluated cases with discrepant scores (L_FCD_ and I_FCD_) to reach a consensus. Finally, these scores were added to obtain a total FCD score (L_FCD_ + I_FCD_) for further analysis. The maximum possible FCD score was 6, where both the lateral and inferior walls were assigned a score of 3, while the minimum score was 0 (both walls were assigned a score of 0).

### Statistical analysis

All statistical analyses were conducted using SPSS 25.0 (IBM) and GraphPad Prism 7 (GraphPad Software). Qualitative data were expressed as frequencies (percentages). Inter-observer agreement from the 2 observers’ independent analyses was tested using Cohen’s kappa test, and the strength of agreement was rated as follows: slight 0.00–0.20, fair 0.21–0.40, moderate 0.41–0.60, good 0.61–0.80, and excellent 0.81–1.00. Proportions of L_FCD_ and I_FCD_ scores were compared between the FCD and control groups using the Pearson chi-squared test, or, when there were fewer than 5 subjects in any cell, using the 2-tailed Fisher exact test. The optimal cut-off value of the FCD score for identifying FCD was determined using receiver operating characteristics (ROC) curve analysis, and the area under the curve (AUC) was calculated. Sensitivity, specificity, and Youden’s index were calculated for each cut-off point. The diagnostic value of the optimal cut-off point was assessed by calculating the positive predictive value (PPV), negative predictive value (NPV), false-negative rate (FNR), false-positive rate (FPR), and accuracy, using surgical finding as the gold standard. *p* values < 0.05 were considered indicative of statistical significance.

## Results

### Demographic and clinical characteristics of the study population

A total of 40 patients (21 females, 19 males) with surgically examined tympanic FC were included. The mean age was 44.3 ± 17.4 years (range 9–80). Regarding laterality, the left ear was involved in 16/40 (40.0%) patients and the right ear was involved in 24/40 (60.0%) patients. All patients underwent middle ear surgery, including mastoidectomy (*n* = 20), tympanoplasty (*n* = 10, type I = 1, type II = 8, type III = 1), stapedotomy (*n* = 6), facial nerve decompression (*n* = 3), or temporal bone partial resection (*n* = 1). Based on the intraoperative findings, 11 (27.5%) and 29 (72.5%) cases were classified as the control and FCD groups, respectively. The mean ages of patients in the control and FCD groups were 46.2 ± 17.3 years and 39.2 ± 17.6 years, respectively.

### Inter-observer agreement

**Fig. 4:**
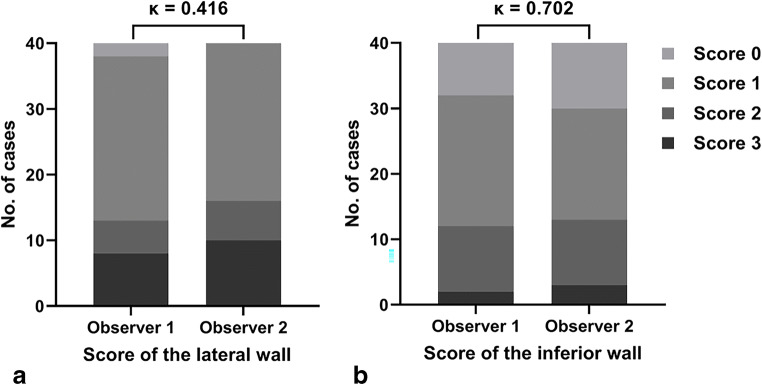
Inter-observer agreement for scores of the lateral (**a**) and inferior (**b**) walls

There was moderate inter-observer agreement with respect to the lateral wall: Cohen’s κ coefficient, 0.416 (95% confidence interval [CI] 0.193–0.639). Good inter-observer agreement was observed for the inferior wall: Cohen’s κ coefficient, 0.702 (95% CI 0.516–0.888) (Table 1 and Fig. 4).

**Table 1:** Inter-observer agreement for L_FCD_ and I_FCD_ scores

Observer 2	Observer 1											
	L_FCD_						I_FCD_					
	0	1	2	3	Cohen’s κ	95% CI	0	1	2	3	Cohen’s κ	95% CI
0	0	0	0	0	0.416	0.193–0.639	7	3	0	0	0.702	0.516–0.888
1	2	20	1	1			1	15	1	0		
2	0	4	1	1			0	2	8	0		
3	0	1	3	6			0	0	1	2		

*L*_*FCD*_ score of the lateral wall, *I*_*FCD*_ score of the inferior wall, *CI* confidence interval

### L_FCD_ and I_FCD_ scores

After discussion, the observers determined L_FCD_ and I_FCD_ scores by consensus. For the lateral wall, the most common appearance was continuous bony covering (L_FCD_ = 3) in the control group (6/11, 54.5%) and discontinuous bony covering with linear deficiency (L_FCD_ = 1) in the FCD group (22/29, 75.9%). For the inferior wall, the most frequently observed imaging appearance was discontinuous bony covering with dotted deficiency (I_FCD_ = 2) in the control group (5/11, 45.5%), and discontinuous bony covering with linear deficiency (I_FCD_ = 1) in the FCD group (15/29, 51.7%) (Table 2).

**Table 2:** Comparison of proportion of L_FCD_ and I_FCD_ scores between the control and FCD groups

Score of the lateral and inferior walls, *n* (%)	Surgical finding		Fisher’s exact value	p^*^
	Control group (*n* = 11)	FCD group (*n* = 29)		
Lateral wall			18.235	< 0.001
Score 0	0 (0.0)	2 (6.9)		
Score 1	1 (9.1)	22 (75.9)		
Score 2	4 (36.4)	3 (10.3)		
Score 3	6 (54.5)	2 (6.9)		
Inferior wall			11.800	0.003
Score 0	0 (0.0)	10 (34.5)		
Score 1	4 (36.4)	15 (51.7)		
Score 2	5 (45.5)	4 (13.8)		
Score 3	2 (18.2)	0 (0.0)		

^*^p value for the Fisher’s exact test

For the L_FCD_ scores, there were 0.0% (0/11), 9.1% (1/11), 36.4% (4/11), and 54.5% (6/11) cases scored 0–3, respectively, in the control group. There were 6.9% (2/29), 75.9% (22/29), 10.3% (3/29), and 6.9% (2/29) scored 0–3, respectively, in the FCD group. The distribution for L_FCD_ score was significantly different between the two groups (Fisher’s exact value = 18.235, *p* < 0.001). For the I_FCD_ score, 0.0% (0/11), 36.4% (4/11), 45.5% (5/11), and 18.2% (2/11) cases were evaluated as scores 0–3, respectively, in the control group. Meanwhile, 34.5% (10/29), 51.7% (15/29), 13.8% (4/29), and 0.0% (0/29) cases in the FCD group were assigned scores 0–3, respectively. The difference for the I_FCD_ score was also statistically significant between the two groups (Fisher’s exact value = 11.800, *p* = 0.003) (Table 2). For both walls, more cases were assigned scores 0 and 1 and less cases were assigned scores 2 and 3 in the FCD group compared to the control group (Table 2).

### FCD score and its diagnostic value

**Fig. 5:**
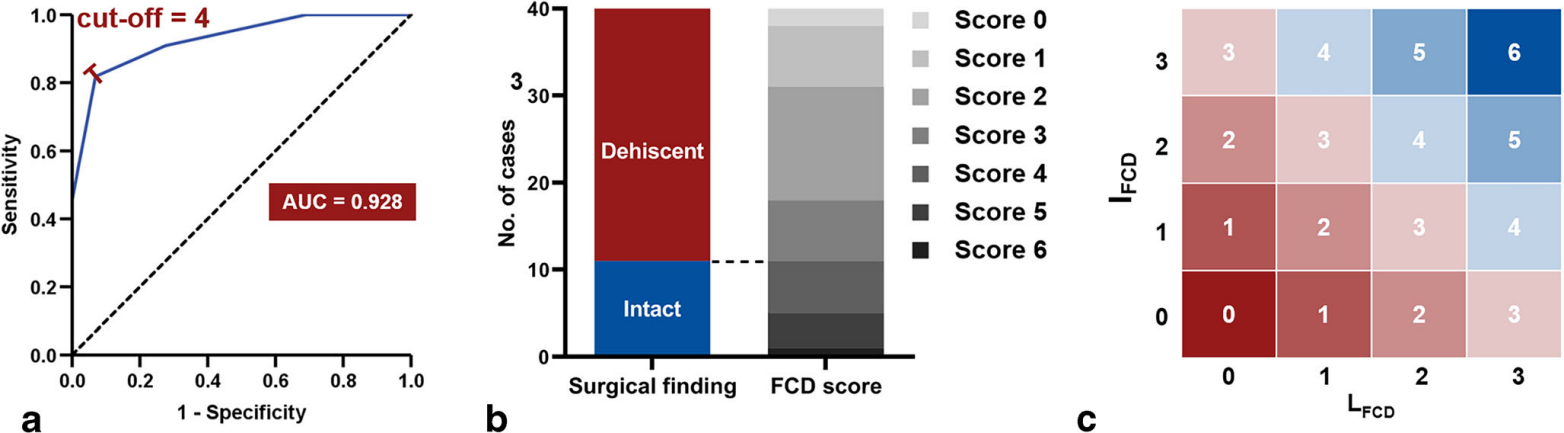
Receiver operating characteristics curve and the area under the curve for FCD score (**a**). The optimal cut-off point is 4, with a sensitivity of 0.82 and specificity of 0.93. There are 2, 7, 13, and 7 cases with FCD scores of 0-3, respectively, thus are considered dehiscent in imaging assessment (**b**). While 6, 4, and 1 cases are assigned FCD scores of 4-6, respectively, hence are considered intact in imaging analysis (**b**). FCD score is the sum of L_FCD_ and I_FCD_. With the cut-off value of 4, 10 combinations of I_FCD_ and L_FCD_ scores are indicative of FCD (red in **c**), and others are considered without FCD (blue in **c**)

As shown in Table 3, the optimal cut-off value (< 4) of the FCD score for detecting FCD was associated with high sensitivity (0.82) and specificity (0.93) with an AUC of 0.928 (Fig. 5). The distribution of the FCD score was as follows: score 0 (*n* = 2), score 1 (*n* = 7), score 2 (*n* = 13), score 3 (*n* = 7), score 4 (*n* = 6), score 5 (*n* = 4), and score 6 (*n* = 1) (Fig. 5). Using the cut-off value of 4, 29, and 11 cases were recognized as with and without FCD, respectively. The PPV and NPV with an FCD score < 4 were 0.82 and 0.93, respectively. The FNR, FPR, and accuracy were 0.18, 0.07, and 0.90, respectively (Table 4).

**Table 3:** Diagnostic performance of various cut-off points of FCD score

FCD score	Sensitivity	Specificity	Youden’s index
< 0	1.00	0.00	0.00
< 1	1.00	0.07	0.07
< 2	1.00	0.31	0.31
< 3	0.91	0.72	0.63
< 4	0.82	0.93	0.75
< 5	0.45	1.00	0.45
< 6	0.09	1.00	0.09

**Table 4:** Diagnostic values of FCD score with a cutoff point of 4

	Surgical finding	
	Control group (*n* = 11)	FCD group (*n* = 29)
Imaging diagnosis, *n* (%)		
Intact (*n* = 11)	9 (81.8)	2 (6.9)
Dehiscent (*n* = 29)	2 (18.2)	27 (93.1)
Diagnostic value		
PPV	0.82	
NPV	0.93	
FNR	0.18	
FPR	0.07	
Accuracy	0.90	

*PPV* positive predictive value, *NPV* negative predictive value, *FNR* false-negative rate, *FPR* false-positive rate

More specifically, an FCD score < 4 included the following scenarios (Fig. 5):

1. L_FCD_ 0+I_FCD_ 0: no bony coverage for both walls.
2. L_FCD_ 0+I_FCD_ 1 or L_FCD_ 1+I_FCD_ 0: no bony coverage for one wall, and discontinuous bony covering with linear deficiency for the other.
3. L_FCD_ 0+I_FCD_ 2, L_FCD_ 1+I_FCD_ 1, or L_FCD_ 2+I_FCD_ 0: no bony coverage for one wall and discontinuous bony covering with dotted deficiency for the other, or discontinuous bony covering with linear deficiency for both walls.
4. L_FCD_ 0+I_FCD_ 3, L_FCD_ 1+I_FCD_ 2, L_FCD_ 2+I_FCD_ 1, or L_FCD_ 3+I_FCD_ 0: no evident bony covering for one wall and with continuous bony covering for the other, or discontinuous bony covering with linear deficiency for one wall and with dotted deficiency for the other.

Other than the combinations above, the tympanic FC was considered intact on U-HRCT images.

**Fig. 6:**
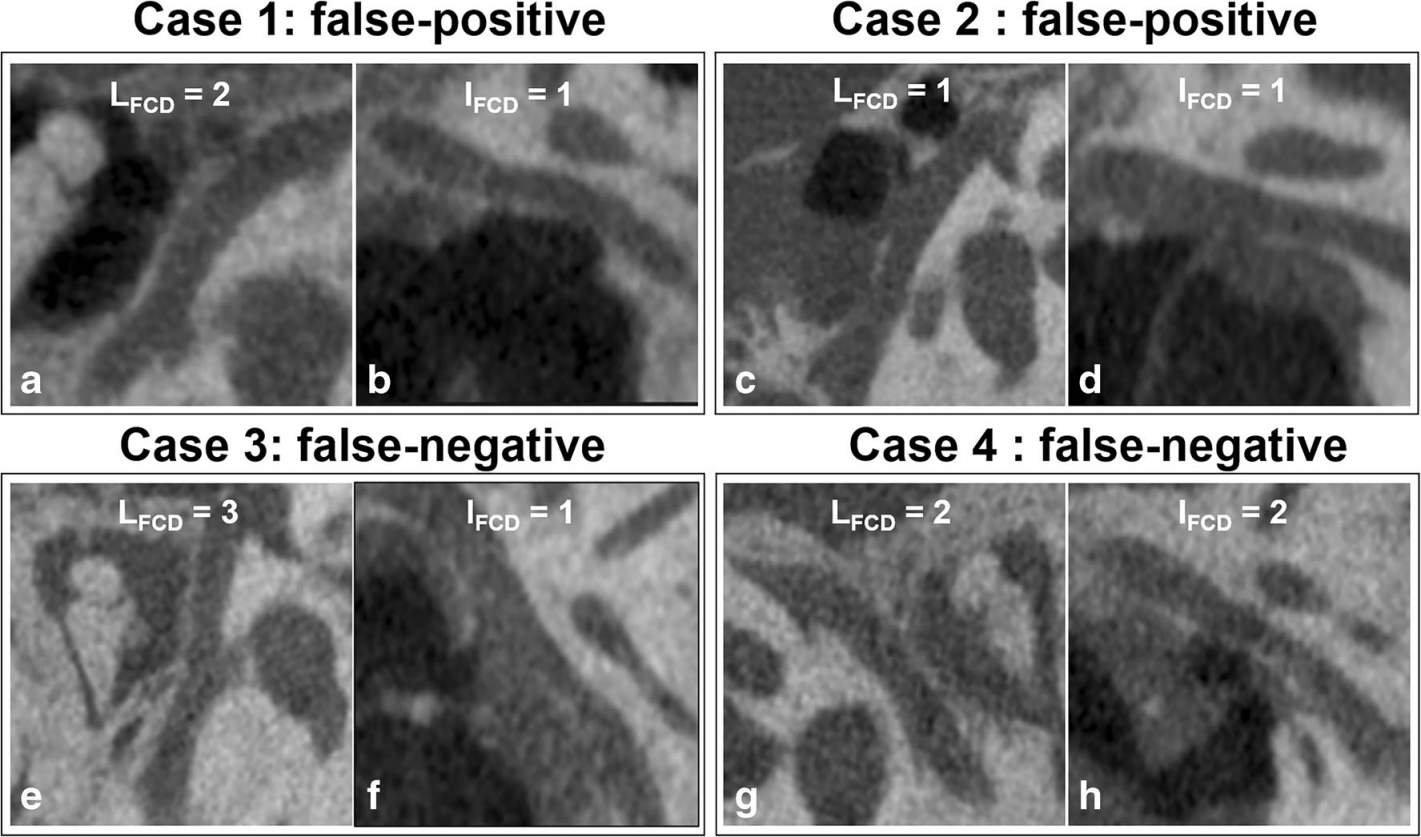
Four misdiagnosed cases using FCD scoring method, among which 2 are false-positive (cases 1 and 2, **a**–**d**) and 2 are false-negative (cases 3 and 4, **e**–**h**)

In addition, 4 cases were misdiagnosed using the FCD scoring method, 2 being false-positive and 2 false-negative (Table 5 and Fig. 6). One false-positive and 2 false-negative cases were diagnosed as COM with/without cholesteatoma, surrounded by extensive inflammatory lesions. The other false-positive case was diagnosed as cholesteatoma.

**Table 5:** Demographic, imaging diagnosis, and surgical findings of misdiagnosed cases

Misdiagnosed cases	Age/sex	Laterality	Disease/surgical procedure	FCD score	Imaging diagnosis	Surgical finding
False-positive	61/F	Right	COM+ cholesteatoma/tympanoplasty	L_FCD_ 2+I_FCD_ 1	Dehiscent	Intact
False-positive	51/F	Right	Cholesteatoma/mastoidectomy	L_FCD_ 1+I_FCD_ 1	Dehiscent	Intact
False-negative	56/F	Right	COM/tympanoplasty	L _FCD_ 3+I _FCD_ 1	Intact	Dehiscent
False-negative	46/F	Left	COM/tympanoplasty	L _FCD_ 2+I _FCD_ 2	Intact	Dehiscent

*COM* chronic otitis media

## Discussion

This study described the detailed CT appearance of the tympanic FC based on U-HRCT with 0.1-mm spatial resolution. We classified the imaging manifestations of the lateral and inferior walls of the tympanic FC into 4 types and found that discontinuous bony covering with linear deficiency was the most common appearance for both walls in cases with FCD, accounting for 75.9% and 51.7% cases, respectively. Moreover, a novel FCD score, consisting of L_FCD_ and I_FCD_, was proposed to identify FCD on U-HRCT images. An FCD score less than 4 was found to be the optimal cut-off value for identifying FCD, with a sensitivity of 0.82 and a specificity of 0.93. Based on the cut-off point, 10 combinations of imaging manifestation were highly indicative of FCD.

The reported prevalence of FCD shows wide variability between clinical studies (6–33.3%) [4–7] and anatomical studies (25–57%) [8, 9], as well as between healthy (51.2%) [3] and otologically diseased populations (11.3%–36.6%) [18, 19]. Owing to its common occurrence, FCD is reckoned as an anatomical variation [20]. However, this anatomical variation may cause problems in pathologic conditions, leading to significant morbidity and occasional mortality [21]. Since the tympanic segment is most commonly found with FCD (taking up 76.2–91%) [8, 18, 22], and the latter lies in proximity to the extension route of cholesteatoma, a dehiscent tympanic FC may be involved by infection, causing severe complications such as facial paralysis [13].

Since the tympanic FC has the thinnest epineural sheath (0.09 mm) [23], the currently used MSCT devices in clinical practice do not allow for a reliable diagnosis of FCD [3, 12, 13]. It is certain that accurate depiction of FC depends on the spatial capability of CT devices. With a slice thickness of 1 mm, the concordance between imaging diagnosis and surgical findings ranged from 42 to 88.2% [4, 24]. The reported sensitivity and specificity of MSCT in identifying FCD were 64.7% and 78.4%, respectively [4]. The discrepancy in diagnostic values can be explained by different settings of CT devices or undetermined CT appearance but is more likely attributable to the thinness of bony covering of the tympanic FC.

CT appearance of FCD and the diagnostic accuracy still remain undetermined. To date, several attempts have been made to describe CT’s appearance. Yetiser et al [13] determined that the tympanic FC was exposed when no bony covering was detected. Tanrivermi et al [14] defined FCD as discontinuity of the bony structure, presenting as a direct connection of the nerve and middle ear space. Arias-Marzán et al [4] considered FCD as an interruption of the bony coating in both coronal and axial planes. Hudson et al [25] quantitatively defined FCD as decreased attenuation at the interface between the FN and tympanic cavity, lacking spiky attenuation.

The majority of previous studies proposed a binary classification of imaging manifestation of the tympanic FC, i.e., lack of bony coating *v.s.* continuous bony covering. Based on observation from U-HRCT images, we added another 2 types: discontinuous bony covering with linear deficiency and discontinuous bony covering with dotted deficiency. Zhang et al [26] classified the tympanic FC based on image quality and imaging appearance as follows: poor image quality with the wall being unidentifiable, poor image quality with the wall being partly displayed, acceptable image quality with the wall edge being blurred, and good image quality with the wall edge being smooth. However, the integrity of the tympanic FC was not discussed in their study. Based on the imaging appearance, we proposed a quantitative FCD scoring method, which, to the best of our knowledge, has not been described in the literature. We also found that majority of the lateral walls (75.9%, 22/29) and more than half of the inferior walls (51.7%, 15/29) presented with discontinuous bony covering with a linear deficiency in the FCD group. With a cut-off value < 4, the FCD scoring method provided high sensitivity and specificity. For both walls, the FCD group showed a greater proportion of no evident bony covering (score 0) and discontinuous bony covering with linear deficiency (score 1), and a smaller proportion of discontinuous bony covering with dotted deficiency (score 2) and continuous bony covering (score 3).

Some studies investigated the anatomic features of FCD itself, and furthermore, examined its relationship with other structures. In the study by Kozerska et al [27], the shape of FCD was reported to range from elliptic, fusiform, to trapezoidal. Tanrivermi et al [14] assessed the association between the second genu angle and the occurrence of FCD and found that in patients with cholesteatoma, cases with FCD had a wider angle than those without FCD. Unlike their studies, we exhibited the value of CT images from a diagnostic point of view, and quantitative measurements were not carried out.

The misdiagnosed cases in our study may be attributed to the display capability of U-HRCT, as well as to the location and shape of FCD. The appearance of linear and dotted deficiency may be due to the image noise, especially in cases with extensive lesions, as shown in Fig. 6. One false-positive and 2 false-negative cases in this study had extensive inflammatory lesions, which may mask the tympanic FC and hamper accurate imaging interpretation. Kozerska et al [27] reported that one-third of FCD involved the inferior wall and was located above and backward to the oval window, but it may also be located on or near the first genu [18, 25]. We proposed the scoring method to assess the lateral and inferior walls on 4 slices from axial or sagittal planes, with the aim of higher reproducibility and to avoid inaccurate evaluation at the margin of FC; however, it may lead to a false-positive result. For example, in cases where FCD occurs in proximity to the first genu but was not explored by surgery. In addition, when FCD was located in the lateral wall but the intact inferior wall was probed by operating surgeons instead, it could also lead to a false-positive reading.

It is noteworthy that the inter-observer agreement of imaging appearance was higher for the inferior wall (Cohen’s κ coefficient 0.702) than for the lateral wall (Cohen’s κ coefficient 0.416). There are several possible explanations. First, some of the tympanic FCs presented with elliptical, tilted shapes on coronal images. The standard axial images, being parallel to the lateral semicircular canal, in such cases may not be perpendicular to the lateral wall of the FC, making an evaluation of the lateral wall more inconsistent compared to the inferior wall, and therefore leading to the moderate inter-observer agreement. Second, the lateral wall showed a more tortuous tract than the inferior wall, with the latter consistently showing a smooth curvy tract in the antero-posterior direction. Although we tried to standardize the observation plane and make the axial plane as parallel to the long axis of the tympanic FC as possible, the results revealed the discrepancy between the two observers, resulting in moderate inter-observer consistency for the lateral wall.

Some limitations of this study should be acknowledged. First, a point-to-point comparison between the imaging and surgical findings was not performed. This was because of its retrospective nature. As a pilot study, we categorized the tympanic FC into dehiscent or intact based on the FCD scoring method, and the size or shape of FCD was not further discussed herein. Second, the small sample size in this study may limit the statistical power of the analysis, and the scoring method needs to be applied in larger populations. Last, we included only two observers, which may have introduced an element of bias. The applicability of the proposed scoring method should be confirmed in a large population of radiologists, and experience-related differences among the observers should be discussed in a future study.

Based on U-HRCT images, the imaging manifestation of the tympanic FC was examined and described on standardized planes and slices by 2 neuroradiologists blinded to surgical findings. The inter-observer agreement was moderate to good for the lateral and inferior walls, respectively. A novel FCD scoring method was proposed and an FCD score less than 4 was found to be the optimal cut-off value with high sensitivity and specificity for detecting FCD, using surgical findings as the gold standard. In addition, we identified 10 combinations of U-HRCT findings that were highly indicative of FCD.

## Supplementary Information

ESM 1 (DOCX 305 kb)

## Abbreviations

AUC: Area under the curve
CI: Confidence interval
COM: Chronic otitis media
FC: Facial canal
FCD: Facial canal dehiscence
FN: Facial nerve
FNR: False-negative rate
FPR: False-positive rate
MSCT: Multislice computed tomography
NPV: Negative predictive value
PPV: Positive predictive value
ROC: Receiver operating characteristics
U-HRCT: Ultra-high-resolution computed tomography

## Declarations

### Guarantor

The scientific guarantor of this publication is Zhenchang Wang.

### Statistics and biometry

No complex statistical methods were necessary for this paper.

### Methodology

• retrospective

• diagnostic study

• performed at one institution
