# Supplementary material for: A novel imaging scoring method for identifying facial canal dehiscence: an ultra-high-resolution CT study
Source: Eur Radiol. 2022 Nov 15;33(4):2830–9. doi: 10.1007/s00330-022-09231-2 (PMC10017601; doi:10.1007/s00330-022-09231-2)
Supplement: Supplementary file 1 — (DOCX 305 kb) [file 330_2022_9231_MOESM1_ESM.docx]

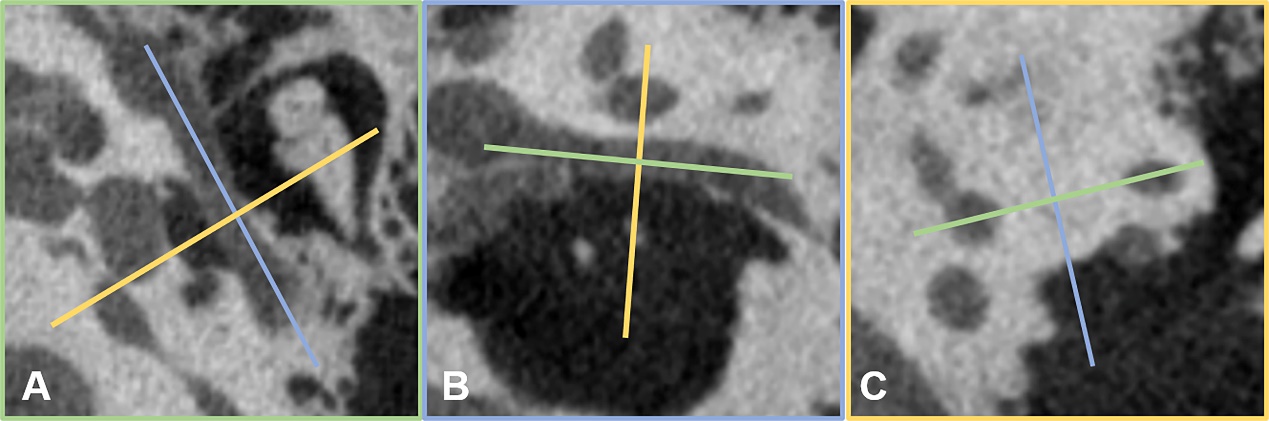


**Supplementary Figure 1.** Standard axial (A), sagittal (B), and coronal (C) planes. The coronal position line (yellow) is perpendicular to the long axis of the tympanic facial canal, and the axial (green) and sagittal (blue) position lines are parallel to the lateral and superior semicircular canals, respectively.
